# Pleural Fluid Resolution Is Associated with Improved Survival in Patients with Malignant Pleural Effusion

**DOI:** 10.3390/life13051163

**Published:** 2023-05-11

**Authors:** Christina R. MacRosty, Amber Wright, Agathe Ceppe, Sohini Ghosh, A. Cole Burks, Jason A. Akulian

**Affiliations:** 1 Section of Interventional Pulmonology and Pulmonary Oncology, Division of Pulmonary Diseases and Critical Care Medicine, University of North Carolina at Chapel Hill, Chapel Hill, NC 27599, USA; 2 Carolina Center for Pleural Disease, University of North Carolina at Chapel Hill, Chapel Hill, NC 27599, USA; 3 Marsico Lung Institute/Cystic Fibrosis Research Center, Department of Medicine, University of North Carolina at Chapel Hill, Chapel Hill, NC 27599, USA; 4 Interventional Pulmonology, Division of Pulmonary and Critical Care Medicine, Allegheny Health Network, Pittsburgh, PA 15222, USA

**Keywords:** malignant pleural effusion, indwelling pleural catheter, spontaneous pleurodesis, pleural disease, resolution of pleural effusion, lung cancer, breast cancer, lymphoma

## Abstract

Malignant pleural effusion is associated with a poor prognosis and, while risk stratification models exist, prior studies have not evaluated pleural fluid resolution and its association with survival. We performed a retrospective review of patients diagnosed with malignant pleural effusion between 2013 and 2017, evaluating patient demographics, pleural fluid and serum composition, and procedural and treatment data using Cox regression analysis to evaluate associations with survival. In total, 123 patients were included in the study, with median survival from diagnosis being 4.8 months. Resolution of malignant pleural fluid was associated with a significant survival benefit, even when accounting for factors such as placement of an indwelling pleural catheter, anti-cancer therapy, pleural fluid cytology, cancer pheno/genotypes, and pleural fluid characteristics. Elevated fluid protein, placement of an indwelling pleural catheter, and treatment with targeted or hormone therapies were associated with pleural fluid resolution. We conclude that the resolution of pleural fluid accumulation in patients with malignant pleural effusion is associated with a survival benefit possibility representing a surrogate marker for treatment of the underlying metastatic cancer. These findings support the need to better understand the mechanism of fluid resolution in patients with malignant pleural effusion as well as the tumor–immune interplay occurring with the malignant pleural space.

## 1. Introduction

Over 150,000 new cases of malignant pleural effusion (MPE) are diagnosed annually, with lung and breast cancer being the most common primary histologies [1]. The etiology of malignant pleural effusion is likely multifactorial and may include tumor emboli to the visceral pleura with seeding of the parietal pleura, direct extension from nearby sites, and hematogenous metastasis [2]. An MPE diagnosis signifies advanced disease and poor prognosis; mean survival from diagnosis in all cancer types ranges from three to twelve months, and averages 74 and 192 days for lung and breast cancer, respectively [3,4,5]. Treatment paradigms focus on the palliation of dyspnea, which can be achieved with serial thoracentesis, placement of an indwelling pleural catheter (IPC), chemical pleurodesis (CP), or surgical interventions such as mechanical pleurodesis [1]. Placement of an indwelling pleural catheter and performance of chemical pleurodesis are recommended for MPE with known or suspected expandable lung in the absence of prior definitive therapy. IPC alone is recommended for patients with nonexpendable lungs, failed pleurodesis, or in cases of loculated effusions, as these lungs are less likely to re-expand to allow for pleural symphysis [6].

Factors associated with decreased survival in patients newly diagnosed with MPE include low pleural fluid (PF) pH, low pleural fluid glucose, high pleural fluid neutrophil count, elevated lactate dehydrogenase (LDH), a high serum neutrophil-to-lymphocyte ratio, primary malignant cell type, and poor performance scores (therefore indicating worse functional status) [2,4,5,7,8,9,10,11,12]. Prognostic scores based on readily available clinical testing can be used to help guide clinical decisions in the treatment of MPE. Both the LENT (lactate dehydrogenase, ECOG, neutrophil-to-lymphocyte ratio, and tumor type) and PROMISE scoring systems have been validated as risk stratification scores to predict survival and help guide clinicians in the care of patients diagnosed with MPE [4,13]. LENT was the first validated risk scoring system for patients with MPE and was shown to be significantly more accurate in predicting survival than ECOG performance status alone. One limitation of the LENT score was the exclusion of treatment data (i.e., chemotherapy) from the scoring system. The PROMISE score was the first prospectively validated prognostic model for MPE that combined clinical and biomarker parameters. Validation of the PROMISE score reported robust accuracy in estimating 3-month mortality in patients with MPE. The PROMISE score also included treatment data (cytotoxic chemotherapy and radiotherapy) prior to diagnosis of MPE and, when compared to LENT, appeared to perform better despite a smaller sample size [4,13]. The clinical utility of these scores in real-time decision-making is limited and often the scores serve as one piece of data within a larger discussion with patients diagnosed with MPE. Most providers that treat malignant pleural effusion continue to use a shared decision-making process focused on patient preference when making recommendations for the treatment of MPE.

These prior studies have provided real insight into MPE, patient survival, and risk stratification. However, they do not evaluate the relationship between available interventions for MPE, the response of MPE to treatment, prognostic factors, and survival. We hypothesize that patients newly diagnosed with MPE who experience resolution of pleural fluid accumulation have longer survival times from the date of MPE diagnosis than those who continue to re-accumulate their malignant effusion. In this study, we evaluated the contribution of previously studied and novel patient factors on survival after MPE diagnosis.

## 2. Materials and Methods

We performed a retrospective chart review of patients with newly diagnosed MPE who presented to our Interventional Pulmonology service between 1 September 2013 and 30 September 2017. Institutional review board approval was obtained prior to study initiation (IRB#17-2478). Patients were identified based on ICD-9 diagnosis codes for MPE. Inclusion criteria were patients with pleural effusion who underwent diagnostic thoracentesis with the Interventional Pulmonary service, a cytology result positive for malignant cells, and age over 18. Exclusion criteria included previous thoracentesis diagnostic for malignancy, negative or indeterminate cytology, age less than 18 years, and/or index thoracentesis by services other than Interventional Pulmonary.

### 2.1. Study Population Data Collected

Data collected included patient demographic information, performance score (ECOG), pleural fluid chemistries and cytology, serum laboratory values, PF PD-L1 testing/status, targetable mutations/fusion protein status (EGFR, EML4-ALK, ROS-1, BRAF, MET, HER2, PIK3CA), indwelling pleural catheter placement, time to resolution of pleural fluid accumulation, systemic treatment information (cytotoxic chemotherapy, targeted/immune/hormonal therapy), and date of death. 

### 2.2. Study Endpoints

The primary aim of this study was to evaluate for an association between the resolution of pleural fluid accumulation and survival in patients diagnosed with MPE. Resolution of PF accumulation for patients undergoing serial thoracentesis was defined as improvement in presenting symptoms and no need for subsequent pleural procedures, and/or no evidence of pleural effusion recurrence on chest imaging. For patients with IPC placement, pleural fluid resolution was defined as improvement in presenting symptoms and IPC drainage of less than 50 mL for three consecutive drainages at least 24 h apart, allowing for removal of the IPC, and without the need for further drainage procedures. Ultrasound and radiographic evidence were not used to confirm pleural symphysis. The secondary goals were to define clinical and laboratory features associated with improved survival. 

### 2.3. Statistical Analysis

Patient clinical and laboratory characteristics were summarized using means ± standard deviations or medians with 25th and 75th quartiles for continuous variables and proportions for categorical variables. Overall survival was calculated in months from the date of MPE diagnosis to the date of death. Surviving patients were censored at the end of the trial date (4 December 2018). Sample size calculation for the primary aim showed a minimum sample size of 72 to detect a hazard ratio of 0.5, assuming a 20% resolution of pleural fluid in the population (power 0.8, alpha 0.05). Each factor was individually evaluated using a Cox Regression Model to determine a single variable association with survival. Results were expressed in terms of a hazard ratio (HR) with an associated 95% confidence interval. It was decided a priori that all variables collected would be evaluated for Inclusion in multivariable regression analysis. Multivariate Cox Regression modeling with stepwise selection was used to evaluate independent associations/effects on survival. We determined statistical significance as *p* < 0.05 in 2-sided hypothesis testing. All analyses were performed using SAS 9.4 software (SAS Inc., Cary, NC, USA).

## 3. Results

### 3.1. Study Population

Data were obtained on 123 patients newly diagnosed with MPE. Seventy-one subjects (58%) were female and the median age of the study population was 64.4 years (IQR 62.3–66.5). Table 1 shows patient demographics and PF characteristics. Pleural fluid analysis revealed an inflammatory lymphocytic exudative process, consistent with previously described MPE studies. Cytologic diagnosis of PF included adenocarcinoma of the lung (37.4%), breast cancer (26.8%), hematologic malignancy (9.8%), urogenital cancer (7.3%), small cell lung cancer (5.7%), and 13% with a primary tumor designated as “other” (including gastrointestinal, thyroid, renal cell carcinoma, melanoma, neuroendocrine tumors, carcinoma of unclear origin, salivary duct adenocarcinoma, and prostate carcinoma). Tumor mutation and/or immune biomarker testing data were available in 104 (84.6%) patients. Actionable tumor mutation and/or immune biomarker positivity (≥50% expression) was identified in 60 of 104 (57.7%) patients tested. 

### 3.2. Predictors of Survival in Patients with Newly Diagnosed MPE

**Figure 1:**
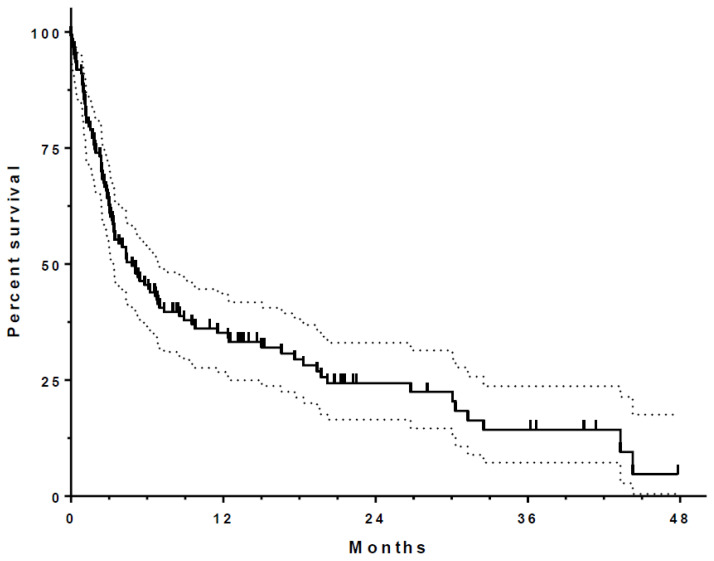
Kaplan–Meier curve showing survival duration of 123 patients included in the study. Dotted lines represent 95% CI.

**Figure 2:**
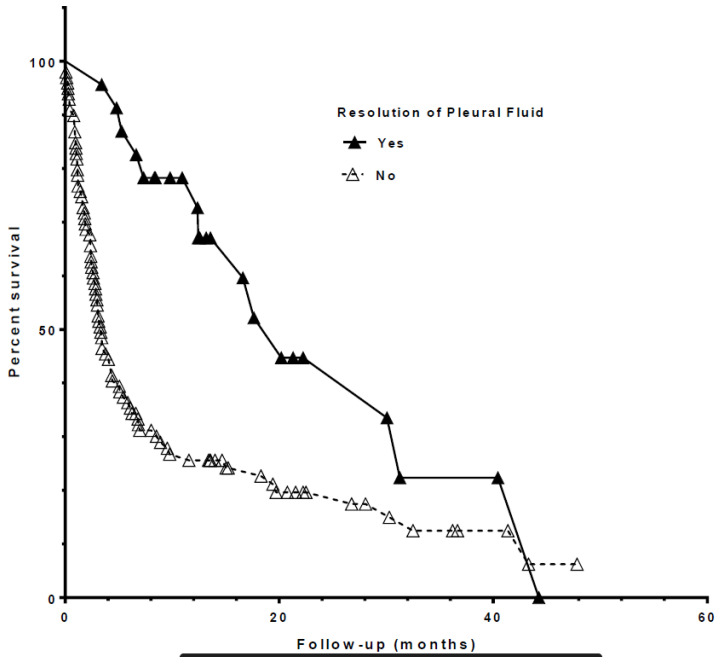
Kaplan–Meier curves showing the relationship between pleural fluid resolution and survival in patients included in the study.

**Figure 3:**
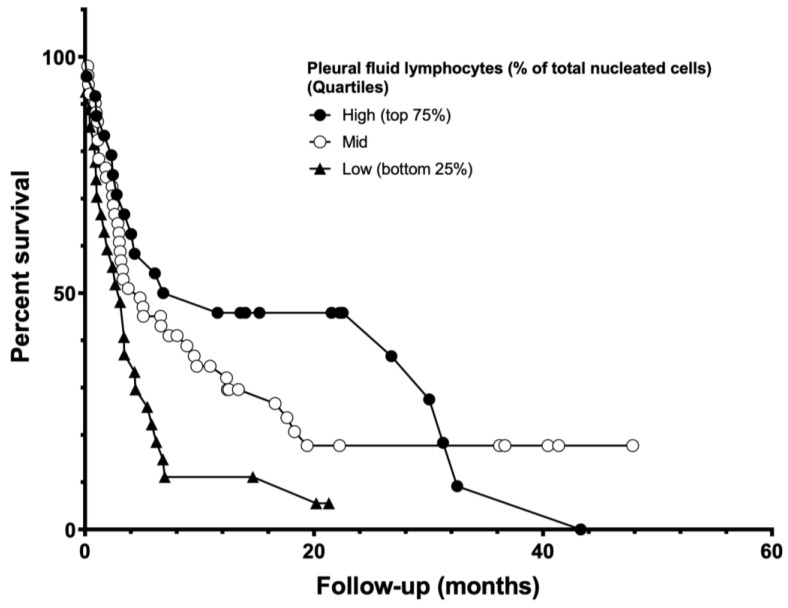
Survival stratified by percent lymphocytes in pleural fluid by quartile.

Median survival from index thoracentesis for all patients was 4.8 months (Figure 1 and Table 2). Patients with a resolution of PF accumulation (*n* = 29) were observed to have an associated survival benefit (HR 0.38, 0.21–0.69, *p* = 0.001; Figure 2). Other factors found to be associated with improved survival included cytotoxic chemotherapy (HR 0.58, 0.38–0.89, *p* = 0.012), higher PF lymphocyte count (Figure 3), and higher PF markers of nutrition (protein and albumin). Elevated PF neutrophil count (HR 1.01, 1.003–1.03, *p* = 0.016) and cytologic cell type of “Other” (thyroid, prostate, and gastrointestinal cancers HR 2.33, 1.28–4.25, *p* = 0.04) were each associated with worse survival after index thoracentesis. Placement of an IPC, the presence of an actionable mutation and/or PD-L1 ≥ 50%, and treatment with targeted, hormonal, or immunotherapy were not associated with an effect on survival. Table 3 shows a univariate analysis of individual variables and their relationship with patient survival.

Causal diagrams and predictor effects of possible confounders are presented in the Supplementary Materials as Supplemental Figure S1 and Supplemental Table S1, respectively. The distribution of baseline variables between the resolution of pleural fluid and the non-resolution of pleural fluid are presented in Supplemental Figure S2.

**Figure 4:**
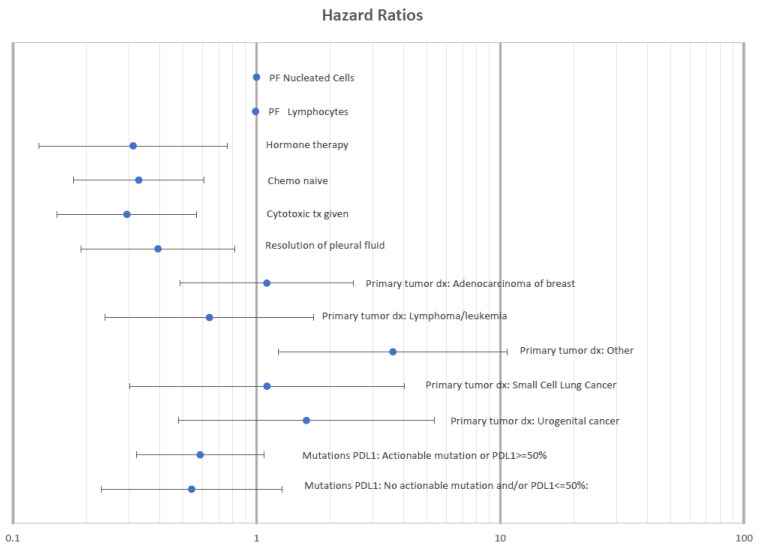
Hazard ratio plot for multivariate regression analysis of factors associated with survival in patients diagnosed with malignant pleural effusion.

Multivariate Cox regression modeling was used to assess the effect of confounding factors on the survival benefit of pleural fluid resolution. The final model confirmed an association between resolution of PF accumulation and improved survival (HR 0.12, 0.03–0.42, *p* = 0.001) when controlling for IPC placement, treatment with cytotoxic chemotherapy, targeted/hormone/immune therapies received, mutation/PD-L1 ≥ 50% status, PF cytologic cell type, PF LDH, PF albumin, PF protein, PF % lymphocyte, and PF % neutrophils. The multivariate model also showed that patients in whom an IPC had been placed had increased hazard ratio for death (HR 6.87, 2.33–20.30, *p* = 0.005), as did those with PF cytology of “Other” (HR 5.20, 1.55–17.45, *p* = 0.008). No association with survival was seen when evaluating different systemic cancer therapies, mutation/PD-L1 status, PF chemistries, or lymphocyte percent of total cell count. Multivariate model results are shown in Figure 4. Please see causal diagram and directed acyclic graphs in the Supplemental Materials.

### 3.3. Predictors of Pleural Fluid Resolution

**Figure 5:**
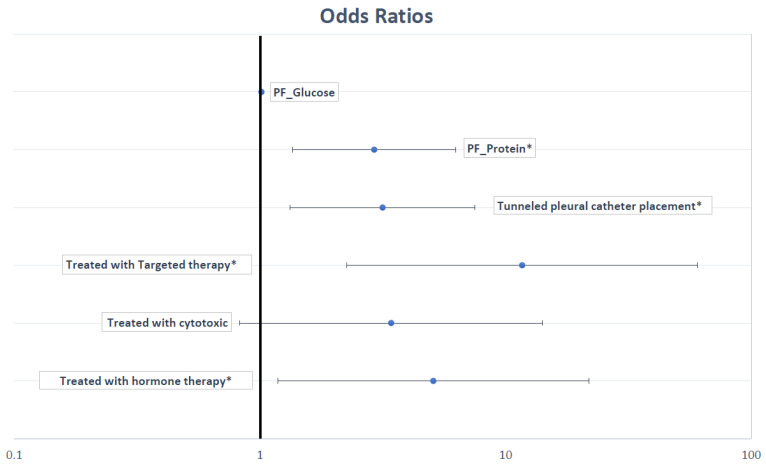
Odds ratio plot for multivariate regression analysis of factors associated with resolution of pleural fluid. * *p* < 0.05.

When evaluating the association between systemic therapy and local therapies on the resolution of PF accumulation, we divided the study population into patients who experienced resolution of PF accumulation versus those that did not. This model (Figure 5) showed that PF protein (OR 2.8, 1.28–6.15, *p* = 0.01), placement of IPC (OR 4.2, 1.46–11.83, *p* = 0.01), treatment with targeted agents (OR 12.33, 2.25–67.76, *p* = 0.004), and/or hormone therapy (5.14, 1.17–22.60, *p* = 0.03) were associated with resolution of PF accumulation. Factors not associated included PF glucose (*p* = 0.39) and treatment with cytotoxic chemotherapy (*p* = 0.11).

## 4. Discussion

The present study was designed to evaluate survival in patients newly diagnosed with malignant pleural effusion who experienced resolution of pleural fluid accumulation and to evaluate the effects of potential confounders on this endpoint. Median survival after diagnosis of malignant pleural effusion in our cohort was similar to prior studies—4.8 and 5 months, respectively [3,14]. Multivariate regression models showed resolution of pleural fluid accumulation to be associated with improved survival.

This study is one of the first to show an independent association between survival and the resolution of pleural fluid accumulation after malignant pleural effusion diagnosis. While novel, this finding is not completely unexpected as it could represent a surrogate for the success of therapy. In multivariate analysis, the resolution of pleural fluid accumulation demonstrated a persistent survival benefit even after controlling for IPC placement, cytotoxic chemotherapy, and targeted and immune cancer therapeutics (TICT) as potential confounders. Analysis of patient/fluid variable effects on the resolution of PF accumulation showed associations with pleural inflammation, the presence of an IPC, and systemic treatment with receptor/protein-specific targeted agents. Despite these findings, the mechanism by which pleural fluid resolution occurs and its relationship with improved survival remain unclear and warrant further study through laboratory, biomarker, clinical, and patient-centered research approaches. The mechanism driving pleural fluid resolution in malignant pleural effusion is likely multifactorial and may include successful treatment of the malignant cell clone driving pleural fluid accumulation, maintenance of visceral to parietal pleural contact in the setting of catheter induced vs. malignant inflammation, and other factors not yet examined. Of note, no data regarding pleural symphysis (true pleurodesis) were available, thus we opted to use the term resolution of PF accumulation. 

The survival function illustrates a rapid drop at the start of post-index thoracentesis follow-up, suggesting the hazard rate for death is highest immediately after the initial diagnosis of MPE. The cause of this apparent high level of mortality after index thoracentesis is unclear. Though we did not perform subgroup analysis on the subset of patients that died soon after MPE diagnosis, these patients may represent those with the most advanced and/or aggressive disease. If true, these patients would likely have had poor performance status, which may have precluded them from receiving definitive therapeutic intervention with either chemotherapy or targeted and immunotherapies. In a study evaluating survival in patients with MPE after their second thoracentesis, Ost et al. reported a median survival of 88 days, which is not dissimilar to our own findings (92 days). The authors report that 37% of patients died before their second pleural procedure and that the mean time to pleural effusion recurrence was 9 days [15]. These findings are consistent with our own and suggest a significant portion of patients presenting with MPE do so in the setting of very poor performance status. While this may explain the post-index thoracentesis hazard rate, this hypothesis warrants further exploration.

When evaluating the effect of the secondary study endpoints on survival, no significant association was found between survival and IPC placement in our univariate analysis. Inclusion of this variable in the multivariate regression model revealed that the placement of an IPC was a strong independent predictor of mortality after controlling for confounding variables. This relationship likely reflects the known effect of advanced stage malignancy on patient survival.

Cytotoxic chemotherapy was the only systemic therapy associated with improved survival during univariate analysis; however, this association was not significant in multivariate regression analysis. Interestingly, TICT were not associated with improved survival in patients with MPE in uni- or multivariate analyses. This is in contrast with previously reported improvements in overall and progression-free survival of patients with EGFR mutation, tyrosine kinase therapy, and MPE, as well as the effect noted in clinical phase III trials of advanced stage malignancy treated with TICT [11,16,17,18,19,20,21,22]. The lack of association in our study is likely due to multiple factors including sample size, use of an aggregate measure (actionable mutations and/or immune biomarker expression), and the MPE of different cytologic cell types, many of which at the time were not routinely treated with targeted or immune modifying agents. Given there was an association between systemic therapy (including targeted agents) and resolution of PF accumulation, the lack of survival benefit may be a blunted signal due to sample size. Further study is needed to determine the role of systemic therapy in the treatment of MPE, including the timing of systemic treatment, the potential toxicity of intrapleural accumulation of chemotherapeutic agents, and the role of interventional therapies such as thoracentesis and indwelling pleural catheters [23].

Each cancer type carries with it a unique profile of prognosis, treatment targets, and responses to therapy and we included assessment of survival after resolution of PF accumulation across cancer types. Clive et al. demonstrated that different cancer types have differing impacts on survival in patients with MPE and assigned a risk stratification score based on these differences [4]. The inclusion of all cancer types reflects a cohort commonly seen within academic and community practices in which referrals are seen from all oncology practices; however, given the difference seen in survival, it is important for providers to evaluate patients based on cancer type when discussing MPE management options.

Univariate modeling suggests MPE with neutrophilic inflammation (elevated neutrophil percent of cell count, LDH, and total protein) found within the pleural space may be associated with decreased survival after index thoracentesis, as reported previously, while PF lymphocytosis is associated with improved survival [8,24]. Neither neutrophil nor lymphocyte counts remained significantly associated with survival in multivariate regression modeling. These findings are consistent with studies evaluating the serum neutrophil:lymphocyte ratio, in which patients with MPE and a higher neutrophilic ratio had decreased survival [4,8,24]. The theorized mechanism driving the survival decrement noted in patients with neutrophilic inflammation has been hypothesized as a neutrophil inhibition of cytotoxic T lymphocyte activation [24,25]. Conversely, improvement in survival reported in MPE patients with high PF lymphocyte count is thought to be the same mechanism as the improvement in survival in patients with elevated levels of tumor-infiltrating lymphocytes. While these are hypothesized to be related, the exact mechanism has not yet been elucidated. This and the theory that the target of activation for immunotherapeutic agents is T-cell activation, priming, and antigen recognition/cytolysis may indicate a potential protective effect of tumor-associated lymphocytosis [26,27,28]. Further characterization of lymphocyte subsets, their function, how they may change during the course of treatment, and their interaction with the tumor cells themselves within the context of the immunologic milieu are required to more fully understand this relationship.

A higher performance score (lower functional status) was significantly associated with an increased risk of death during univariate analysis. This would indicate that lower overall fitness is associated with shorter survival, which has been consistently demonstrated in patients with MPE [3,4,5,10,29,30]. The effect of performance score on survival is likely a surrogate for overall morbidity at the time of index thoracentesis. Despite these strong associations seen during univariate analysis, PS was not included in the multivariate regression due to limited data availability and subsequent model fit.

The findings presented in this manuscript are complementary to the results of the recently published LENT and PROMISE risk scoring systems [4,13]. In both studies, the authors were able to produce robust risk assessment tools by which to stratify patients with MPE. Many of our study’s findings were consistent with these and other publications evaluating the impact of patient and laboratory factors on survival in MPE. Our manuscript differs as it shows an association between improved survival and the resolution of PF accumulation. In addition, ours is the first to include TICT in the survival regression analysis. This is particularly important as TICT have become first-line anti-cancer therapies across a growing number of cell lines (particularly lung, breast, and colorectal cancers as well as melanoma).

There are several limitations to this study. Although multivariate regression methods were used to account for many potential confounding variables, residual confounding factors may yet have affected the presented results. The retrospective design of our study is a limitation since the treatment of MPE was not performed according to a pre-specified protocol and the heterogeneity of treatments could act as a confounding factor. Another limitation is the definition of pleural fluid resolution without direct evidence of pleural symphysis, which did not allow for the evaluation of the formation of pleural loculations as a cause of PF resolution. This may confound our results. However, our practice is to ultrasound the pleural space when IPC output is less than 50 mL for three consecutive drainages to ensure there are no loculations requiring intrapleural fibrinolytic therapy. While complication rates of pleural procedures at our institution are in line with those reported in the literature, we did not collect these data or data on hospital/ICU admissions, which may confound results. Furthermore, sample size variations for individual factors analyzed, resultant sample heterogeneity, and the number of predictors analyzed may play a limiting role in the precision of our results and/or inclusion/exclusion of specific variables into the regression models.

Despite these limitations, we present, for the first time, resolution of PF accumulation as an independent predictor of survival in patients diagnosed with MPE and the factors associated with this event. Further study is needed to assess factors that directly impact the resolution of PF accumulation and if these can predict not only survival but optimal management of MPE. In addition, this study re-demonstrated the roles nutrition status and overall fitness continue to play in survival after diagnosis of MPE, further illustrating the need for a multidisciplinary approach to cancer treatment. Further investigation into the mechanism behind PF accumulation and resolution as well as a more detailed understanding of the interplay of tumor-specific immunity and MPE is needed to understand its association with patient survival. These data are and will be key in developing a more nuanced approach to evaluating MPE patient outcomes and management decisions. The data in the manuscript demonstrate the need for future prospective studies of survival in patients with MPE and the interplay between survival and underlying mechanisms that lead to the development and/or resolution of MPE.

## 5. Conclusions

Malignant pleural effusion indicates advanced disease and is associated with poor survival in all cancer types. Our study is one of the first to demonstrate a potential survival benefit in patients undergoing treatment for their underlying cancer who have a resolution of malignant pleural effusion formation. Whether this survival benefit represents a true benefit or a surrogate to treatment response remains to be seen and requires further study, especially in the evolving era of targeted and immunotherapies.

## Figures and Tables

**Table 1:** Collected patient demographics and pleural fluid data.

Patient Demographic Variables	N	
Age at diagnosis (years)	123	64.4 (62.3–66.5) ^†^
Female	123	71 (58%)
ECOG performance score ^^^	58	0 (*n* = 10; 17.2%)
		1 (*n* = 28; 48.2%)
		2 (*n* = 12; 20.7%)
		3 (*n* = 5; 8.6%)
		4 (*n* = 3; 5.2%)
History of tobacco use	122	72 (59%)
IPC placed	123	63 (51.2%)
**Pleural fluid values**		
pH	25	7.5 (7.4–7.5) ^†^
% eosinophils	74	0 (0–1.0) *
% neutrophils	100	7.5 (2.0–18.5) *
% monocytes/macrophages	102	10.5 (5.0–20.0) *
% lymphocytes	103	43 (18.0–68.0) *
Glucose (mg/dL)	100	93.5 (77.5–113.0) *
Protein (g/dL)	104	4 (3.8–4.2) ^†^
lactate dehydrogenase (U/L)	107	760 (446.0–1411.0) *
Presence of mutation or PD-L1 ≥ 50%	104	60 (57.7%)

Data are presented as absolute number and percentage. ^†^ Mean (95% CI), * Median 25th–75th percentile interquartile range (IQR) values. ^^^ ECOG performance score presented with absolute numbers and percent per category.

**Table 2:** At-Risk Table from Kaplan–Meier Survival Curve Data.

	Spontaneous Pleurodesis = N	Spontaneous Pleurodesis = Y
Number of rows	123	123
rows with impossible data	1	0
censored subjects	18	10
deaths/events	81	13
Median survival (months)	3.3333	20.2

**Table 3:** Univariate regression analysis of patient and pleural fluid factors associated with survival in patients diagnosed with malignant pleural effusion.

Variable	Hazard Ratio (95% CI)	*p* Value
Resolution of pleural fluid accumulation	0.382 (0.2112–0.688)	0.0014
Intrapleural catheter placed	1.231 (0.819–1.849)	0.317
Performance score	1.714 (1.252–2.347)	0.0008
Actionable target and/or PD-L1 ≥ 50%	0.633 (0.399–1.004)	0.0518
Systemic cancer treatment given	0.175 (0.098–0.31)	<0.0001
Cytotoxic therapy	0.579 (0.378–0.885)	0.0116
Hormone therapy	0.619 (0.361–1.064)	0.0824
Targeted therapy	0.632 (0.376–1.06)	0.0822
Immunotherapy	0.782 (0.448–1.364)	0.3859
PF Cytology Group—Breast Cancer	0.755 (0.452–1.33)	
PF Cytology Group—Lymphoma/Leukemia	0.876 (0.387–1.981)	
PF Cytology Group—Non-small Cell Lung Cancer	0.843 (0.258–2.76)	0.041
PF Cytology Group—Small Cell Lung Cancer	1.7 (0.662–4.366)	
PF Cytology Group—Urogenital Cancer	1.287 (0.594–2.791)	
PF Cytology Group—Other Cancer	2.331 (1.278–4.249)	
PF LDH (per 100 units)	1.011 (1.001–1.022)	0.0373
PF Protein	0.738 (0.589–0.924)	0.0081
PF Albumin	0.44 (0.279–0.693)	0.0004
PF % lymphocytes	0.988 (0.97–0.997)	0.0038
PF % neutrophils	1.014 (1.003–1.025)	0.0155

## Data Availability

The data presented in this study are available in the article figures.

## Supplementary Materials

The following supporting information can be downloaded at: https://www.mdpi.com/article/10.3390/life13051163/s1, Supplemental Figure S1. Causal Diagram for Variables Selected for Models; Supplemental Figure S2. Predictor Effect of Possible Confounders; Supplemental Table S1. Distribution of Baseline Variables Between Groups with Resolution of Pleural Fluid and Non-resolution of Pleural Fluid.
