# Supplementary material for: Pleural Fluid Resolution Is Associated with Improved Survival in Patients with Malignant Pleural Effusion
Source: Life (Basel). 2023 May 11;13(5):1163. doi: 10.3390/life13051163 (PMC10223971; doi:10.3390/life13051163)
Supplement: Supplementary file 1 [file life-13-01163-s001.zip › life-2203999-supplementary.pdf]

Supplemental Figure S1. Causal Diagram for Variables Selected for Models

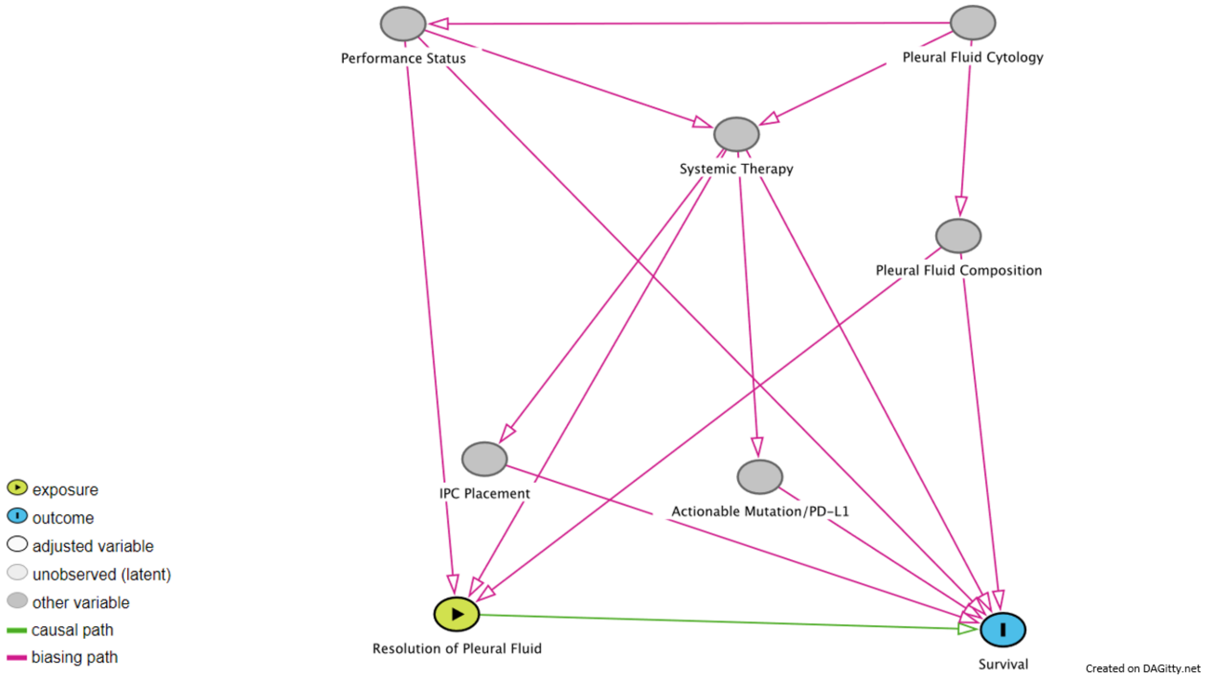

Supplemental Figure S2. Predictor Effect of Possible Confounders

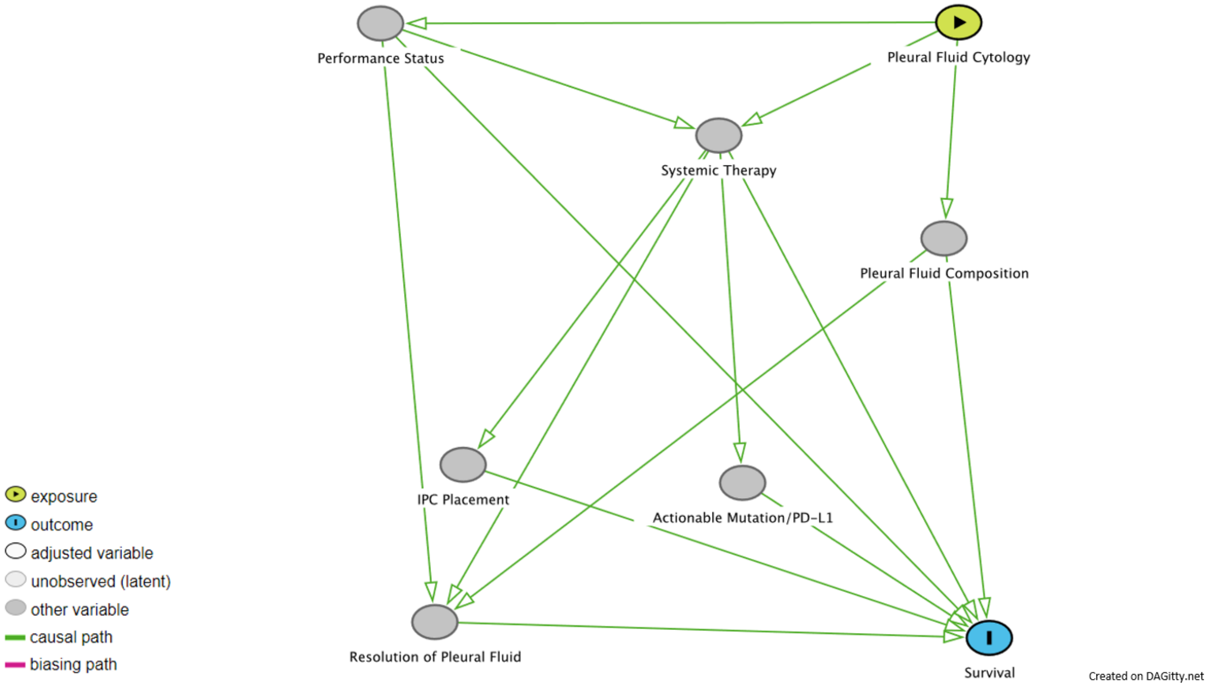

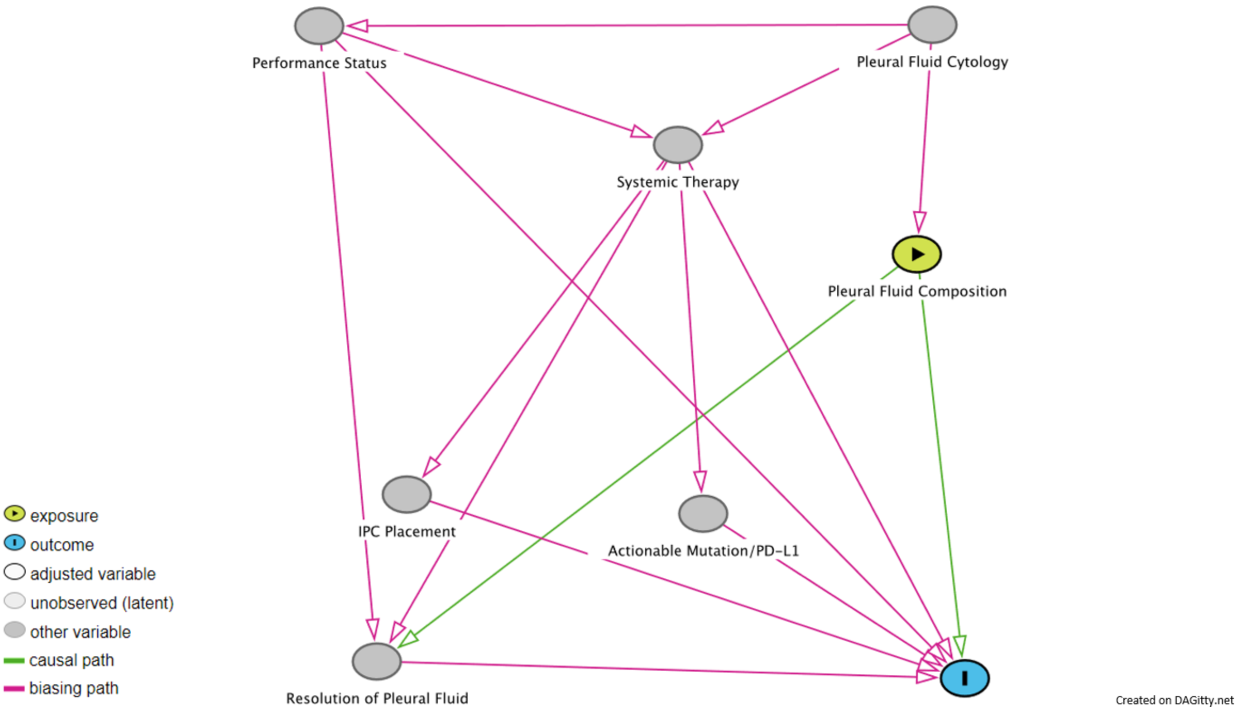

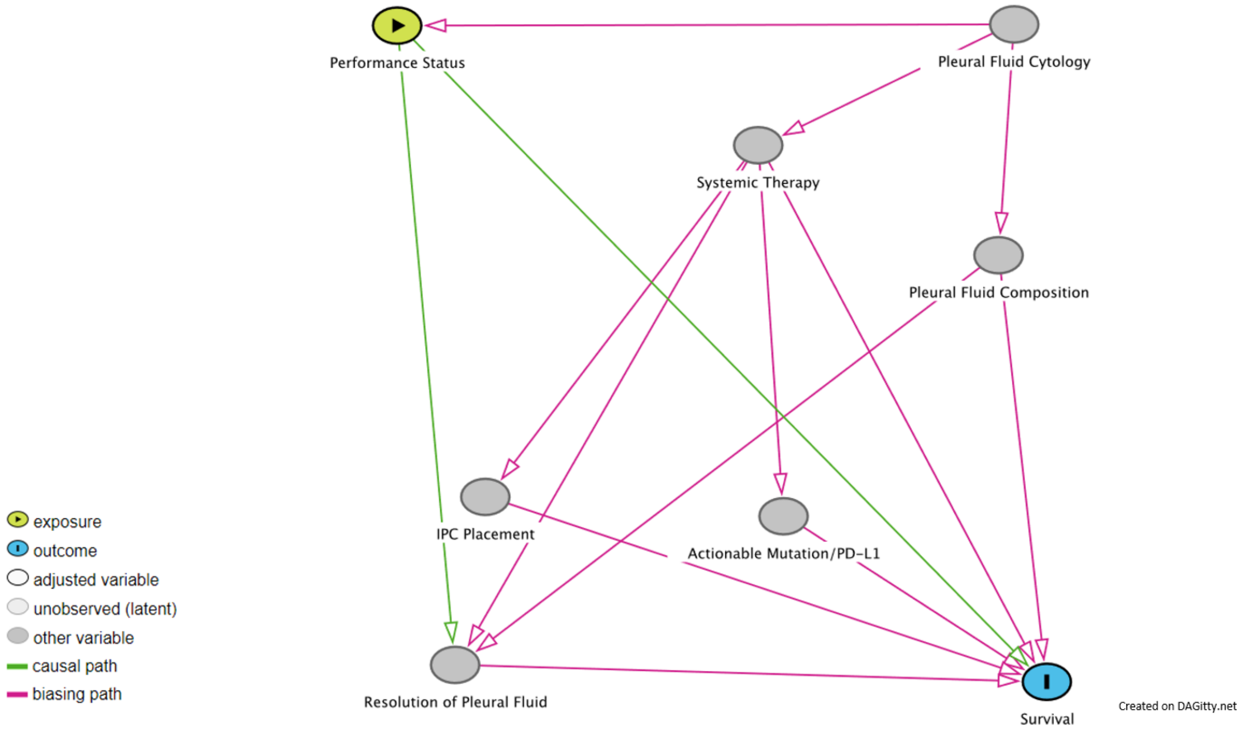
Performance Status
Pleural Fluid Cytology
Systemic Therapy
Pleural Fluid Composition
IPC Placement
Actionable Mutation/PD-L1
Resolution of Pleural Fluid
Survival
exposure
outcome
adjusted variable
unobserved (latent)
other variable
causal path
biasing path
Created on DAGitty.net

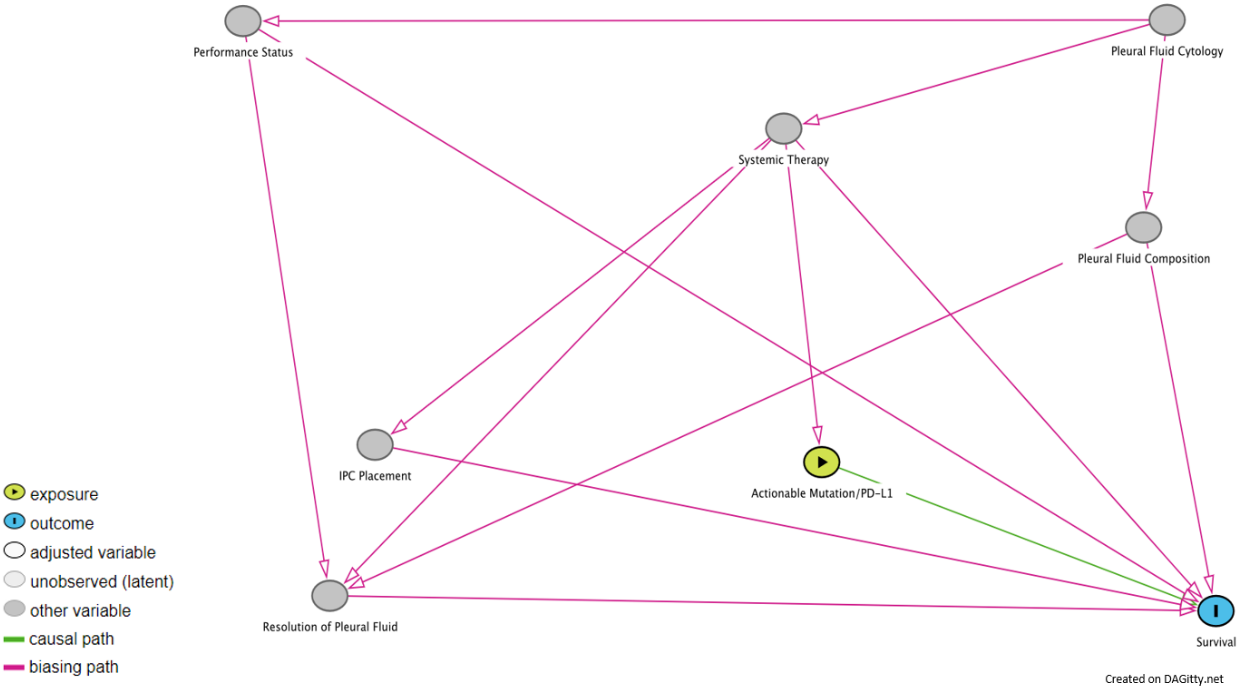
Performance Status
Pleural Fluid Cytology
Systemic Therapy
Pleural Fluid Composition
IPC Placement
Actionable Mutation/PD-L1
Resolution of Pleural Fluid
Survival
exposure
outcome
adjusted variable
unobserved (latent)
other variable
causal path
biasing path
Created on DAGitty.net

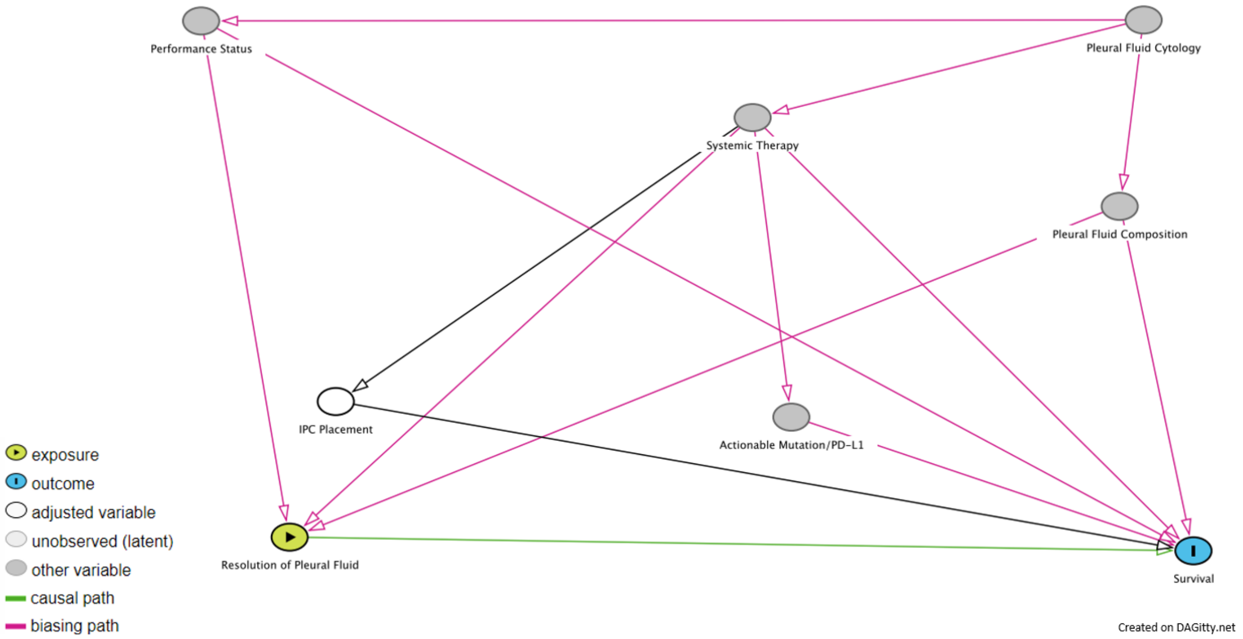

Performance Status
Pleural Fluid Cytology
Systemic Therapy
Pleural Fluid Composition
IPC Placement
Actionable Mutation/PD-L1
Resolution of Pleural Fluid
Survival
exposure
outcome
adjusted variable
unobserved (latent)
other variable
causal path
biasing path
Created on DAGitty.net

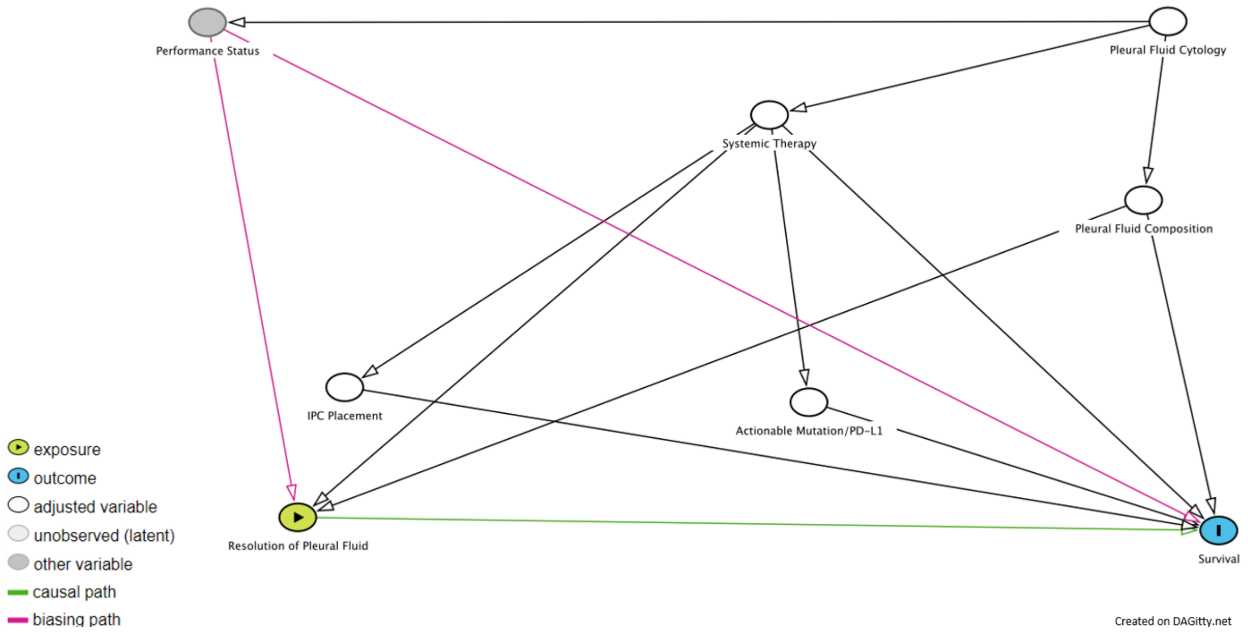

Performance Status
Pleural Fluid Cytology
Systemic Therapy
Pleural Fluid Composition
IPC Placement
Actionable Mutation/PD-L1
Resolution of Pleural Fluid
Survival
exposure
outcome
adjusted variable
unobserved (latent)
other variable
causal path
biasing path
Created on DAGitty.net

Supplemental Table S1. Distribution of Baseline Variables Between Groups with Resolution of Pleural Fluid and Non-resolution of Pleural Fluid.

	PF Resolution		No PF Resolution	
**Parameter**	**Frequency**	**Percent**	**Frequency**	**Percent**
Chemotherapy naive	12	52.17	49	49
Chemotherapy non-naive	11	47.83	51	51
Cytotoxic therapy given	17	73.91	65	65
Cytotoxic therapy not given	6	26.09	35	35
Hormone therapy given	6	26.09	19	19.39
Hormone therapy not given	17	73.91	79	80.61
IPC placed	23	100	40	40
IPC not placed	.	.	60	60
Immunotherapy given	6	26.09	13	13
Immunotherapy not given	17	73.91	87	87
Actionable Mutation	15	65.22	42	52.5
No Actionable Mutation	2	8.7	11	13.75
No mutation detected	6	26.09	27	33.75
PD-L1 50% or greater	3	60	3	33.33
PD-L1 less than 50%	2	40	6	66.67
PF Cytology Adenocarcinoma of breast	7	30.43	23	23
PF Cytology Lymphoma/leukemia	3	13.04	9	9
PF Cytology Non Small Cell Lung Cancer	1	4.35	3	3
PF Cytology Other	2	8.7	15	15
PF Cytology Small Cell Lung Cancer	.	.	5	5

PF Urogenital cancer	.	.	11	11
PF Adenocarcinoma of Lung	10	43.48	34	34
Pleurodesis performed	.	.	5	5.05
Pleurodesis not performed	23	100	94	94.95
Primary tumor diagnosis Adenocarcinoma of breast	8	34.78	25	25
Primary tumor diagnosis Lymphoma/leukemia	3	13.04	9	9
Primary tumor diagnosis Other	1	4.35	15	15
Primary tumor diagnosis Small Cell Lung Cancer	1	4.35	6	6
Primary tumor diagnosis Urogenital cancer	.	.	9	9
Primary tumor diagnosis Adenocarcinoma of lung	10	43.48	36	36
Radiation therapy naive	16	69.57	64	64.65
Radiation therapy non-naive	7	30.43	35	35.35
Radiation therapy given	12	52.17	47	47.96
Radiation therapy not given	11	47.83	51	52.04
Sex	15	65.22	56	56
Sex	8	34.78	44	44
Currently Smoking	1	4.35	11	11.11
Prior Smoking	11	47.83	49	49.49
Never Smoked	11	47.83	39	39.39
Systemic cancer therapy given	23	100	82	82
Systemic cancer therapy not given	.	.	18	18

Targeted therapy given	8	34.78	18	18
Targeted therapy not given	15	65.22	82	82
